# The Foodborne Transmission of Hepatitis E Virus to Humans

**DOI:** 10.1007/s12560-021-09461-5

**Published:** 2021-03-18

**Authors:** Samantha Treagus, Conal Wright, Craig Baker-Austin, Ben Longdon, James Lowther

**Affiliations:** 1 Biosciences, College of Life and Environmental Sciences, University of Exeter, Penryn Campus, Penryn, Cornwall UK; 2 Seacorp Technologies Limited, Bournemouth, UK; 3 Centre for Environment Fisheries and Aquaculture Science, Barrack Road, Weymouth, Dorset DT4 8UB UK

**Keywords:** Hepatitis E virus, Foodborne, Zoonotic, Transmission, Meat, Shellfish

## Abstract

Globally, Hepatitis E virus (HEV) causes over 20 million cases worldwide. HEV is an emerging and endemic pathogen within economically developed countries, chiefly resulting from infections with genotype 3 (G3) HEV. G3 HEV is known to be a zoonotic pathogen, with a broad host range. The primary source of HEV within more economically developed countries is considered to be pigs, and consumption of pork products is a significant risk factor and known transmission route for the virus to humans. However, other foods have also been implicated in the transmission of HEV to humans. This review consolidates the information available regarding transmission of HEV and looks to identify gaps where further research is required to better understand how HEV is transmitted to humans through food.

**Supplementary Information:**

The online version contains supplementary material available at 10.1007/s12560-021-09461-5.

## Introduction

Globally, hepatitis E virus (HEV), of the family *Hepeviridae*, is considered the most common cause of acute viral hepatitis. There were an estimated 20 million infections worldwide annually in 2005 from genotype 1 (G1) and 2 (G2) HEV combined (Rein et al. 2012), and 44,000 recorded fatalities due to the virus in 2015 (World Health Organisation 2019). Generally, HEV causes an acute, self-limiting infection which resolves within a few weeks; however, in some persons (such as the immunocompromised) it can cause chronic infections, fulminant hepatitis (acute liver failure) and extrahepatic manifestations (infections in other organs), which can be fatal. Table 1 shows a summary of the pattern of infection of the different HEV genotypes, demonstrating the variable clinical manifestations and factors such as average age of infection and gender, where these are known. There are limited data to be able to estimate the number of infections worldwide, but with a high level of asymptomatic infections seen in numerous outbreaks it is probable that more infections occur worldwide than estimated in 2005 (Guillois et al. 2015; Yin et al. 2019), especially considering that this estimate was made only considering G1 and G2.

**Table 1:** Pattern of infection for the different genotypes of HEV, adapted from Centers for Disease Control and Prevention (2020)

Genotype	Transmission in humans?	Transmission routes	Geographical distribution pattern	Extrahepatic manifestations	Age groups at higher risk	Gender more commonly affected	Lethality
1	Yes	Faecal-oral; waterborne; blood transfusion; organ donation	Economically developed and developing countries	Pancreatic	Differs by country^a,b^	Differs by country^a,b^	0.5–1%^c^; 20% in pregnant women^d,e,f^
2	Yes	Faecal-oral; waterborne; blood transfusion; organ donation	Economically developing countries	Unknown	Young adults	Unknown	0.5–1%^c^
3	Yes	Foodborne; blood transfusion; organ donation	Economically developed and developing countries	Chronic infections in immune-compromised patients. Neurological, haematological, immunological and renal manifestations^g^	Older adults (> 40 years)	Males	0.5–1%^c^
4	Yes	Foodborne; blood transfusion; organ donation	Economically developed and developing countries	Unknown	Young adults	Possibly males (limited data)^h^	0.5–1%^c^
5	No	Faecal-oral	Unknown	Unknown	Unknown	Unknown	Unknown
6	No	Faecal-oral	Unknown	Unknown	Unknown	Unknown	Unknown
7	Yes	Foodborne; Faecal-oral; blood transfusion?	Unknown	Unknown	Unknown	Unknown	Unknown
8	No	Faecal-oral	Unknown	Unknown	Unknown	Unknown	Unknown

^a^Pathak and Barde (2017)

^b^Spina et al. (2017)

^c^Peron et al. (2007)

^d^Kumar et al. (2017)

^e^Jin et al. (2016)

^f^Kamar et al. (2014)

^g^Horvatits et al. (2019)

^h^Mizuo et al. (2005)

**Fig. 1:**
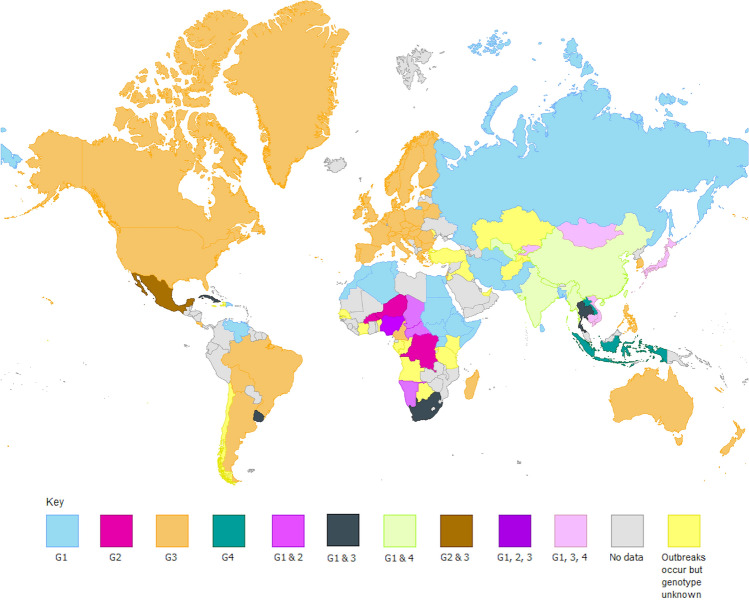
The geographical distribution of HEV genotypes 1–4. This figure shows the genotypes of HEV which are endemic to each country, where enough data were available. For graphs which are compatible with the conditions protanopia, deuteranopia and achromatopsia please see online resources 1 and 2. Maps created in ArcMap using the World Countries (generalised) layer package by esri_dm and visualised in GIMP

HEV was once thought only to be endemic to certain economically developing countries within Asia and Africa, but research over the past decade has highlighted the emergence of HEV within higher income countries. The virus spreads through a faecal-oral route, making it easily transmissible through faecally contaminated water. Indeed, this is thought to be the main transmission route within China, where G1 and genotype 4 (G4) HEV are dominant (Wang et al. 2001). However, it is also possible for the virus to be transmitted through foodstuffs such as pork, due to the ability for some HEV genotypes to infect non-human animals. Currently, the virus is classified into eight genotypes, with G1–4 and genotype 7 (G7) capable of infecting humans. There is, however, a diverse host range for the different genotypes, with G1 and G2 generally only infecting humans and non-human primates, but genotypes 3–8 infecting many other animals, such as pigs, deer, camels, rabbits, and dolphins. A summary of the different host species of HEV was published recently (Kenney 2019), and is summarised briefly in Table 2. Genotype 3 (G3) HEV has been found to be the most geographically diverse of the viruses thus far (Pérez-Gracia et al. 2015), and is the genotype which has emerged in the past 2 decades in many developed countries. The geographical distribution of genotypes 1–4 can be seen in Fig. 1.

**Table 2:** Summary of HEV host species by genotype adapted from Kenney (2019)

Genotype	Hosts identified (common names)	Species infected
1	Humans, Chimpanzees, Monkeys, Horses	*Homo sapiens, Pan troglodytes, Chlorocebus sabaeus*^a^*, Chlorocebus pygerythrus*^a^*, Erythrocebus patas*^a^*, Macaca mulatta, Macaca radiata, Macaca fascicularis, Semnopithecus entellus, Aotus trivirgatus*^a^*, Saguinus mystax mystax*^a^*, Saimiri sciureus*^a^*, Equus caballus ferus*
2	Humans, Monkeys	*Homo sapiens, Chlorocebus pygerythrus*^a^*, Erythrocebus patas*^a^*, Macaca mulatta, Macaca fascicularis, Aotus trivirgatus*^a^*, Saguinus mystax mystax*^a^*, Saimiri sciureus*^a^
3	Humans, Monkeys, Hares and Rabbits, Rats, Minks, Mongooses, Pigs, Goats and Sheep, Deer, Dolphins, Horses, Vultures	*Homo sapiens, Erythrocebus patas, Macaca mulatta, Macaca fascicularis, Macaca fuscata, Lepus europaeus, Oryctolagus cuniculus domesticus, Rattus norvegicus, Neovison vison, Herpestes javanicus, Sus scrofa, Sus scrofa domestica, Capra hircus aegagrus, Ovis aries orientalis, Cervus elaphus, Cervus nippon, Capreolus, Tursiops truncatus, Equus africanus, Equus caballus ferus, Gyps himalayensis*
4	Humans, Monkeys, Gerbils, Dogs, Bears, Leopards, Pigs, Cows, Goats, Deer, Cranes, Pheasants	*Homo sapiens, Macaca fascicularis, Macaca mulatta, Meriones unguiculatus*^a^*, Canis lupus familiaris, Ursus thibetanus, Neofelis nebulosa, Sus scrofa, Sus scrofa domesticus, Bos taurus primigenius, Bos grunniens, Capra hircus aegagrus, Ovis aries orientalis, Cervus nippon, Elaphodus cephalophus, Muntiacus reevesi, Balearica regulorum, Lophura nycthemera*
5	Monkeys, Pigs	*Macaca fascicularis, Sus scrofa*
6	Pigs	*Sus scrofa*
7	Humans, Camels	*Homo sapiens, Camelus dromedarius,*
8	Camels	*Camelus bactrianus*

^a^Infections instigated through experimental conditions

**Fig. 2:**
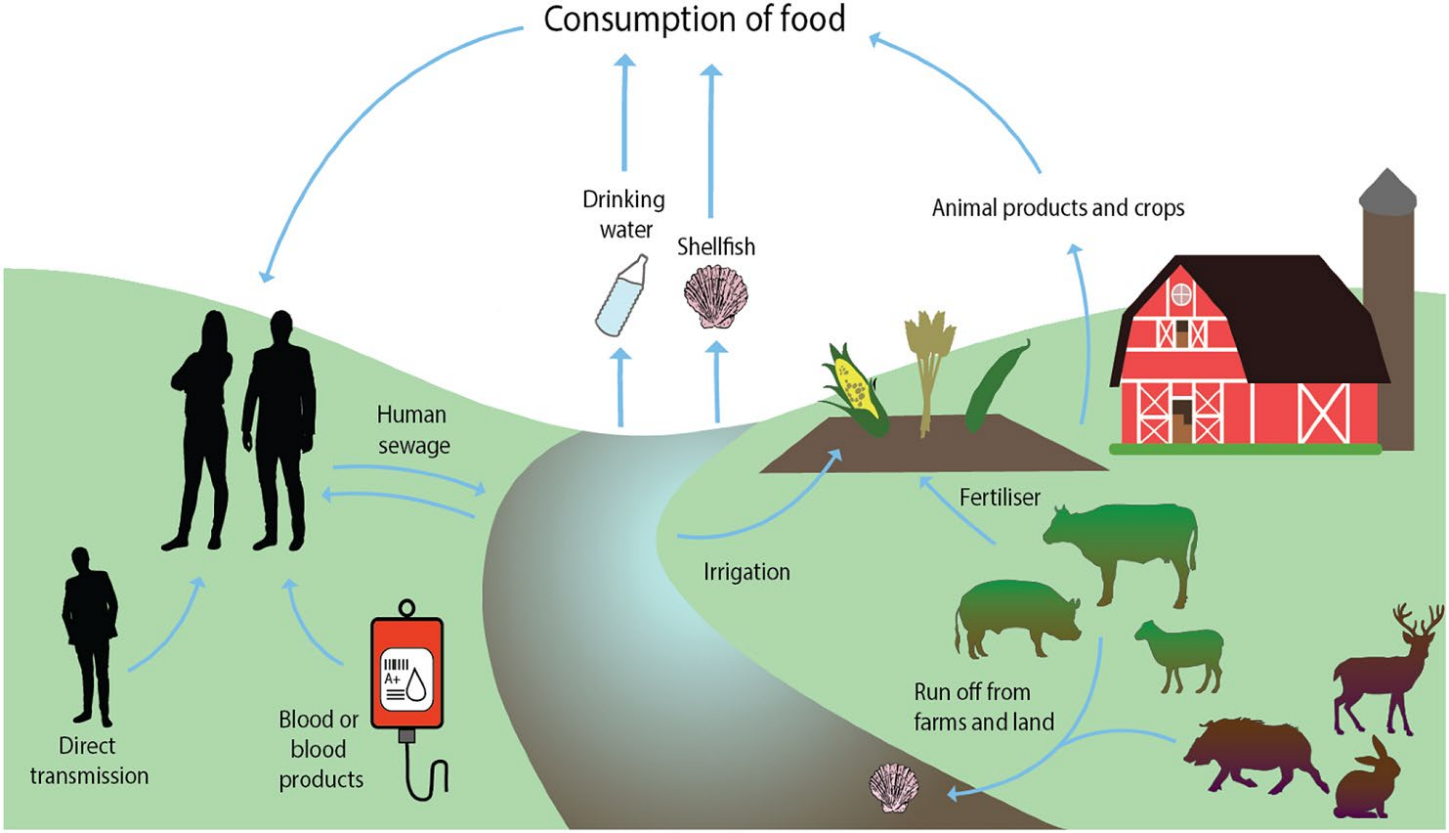
Theoretical and confirmed transmission routes of HEV. The figure shows confirmed and theoretical routes of HEV transmission to humans. The theoretical routes of transmission include HEV infections contracted from the consumption of shellfish, sheep, and cows, as well as crops and drinking water, as no confirmed outbreaks from these sources have yet been identified. Illustration created using Adobe Illustrator and edited using GIMP

G3 HEV is thought to be spread primarily through the consumption of undercooked pork products from infected pigs; however, it is unknown if all transmission routes to humans have been identified. The current theories and known routes of transmission can be seen in Fig. 2. This review will discuss theoretical routes of HEV transmission to humans through foodstuffs and identify areas which require further research for better understanding of the virus.

## HEV in Pigs and Pork Products

Over the past 2 decades, evidence has accumulated implicating pigs and other animals in the zoonotic transmission of G3 HEV to humans (Tei et al. 2003; Guillois et al. 2015; Lhomme et al. 2013; Rivero-Juarez et al. 2017). In 1998, it was shown that a HEV strain isolated from an acute HEV patient in the USA was capable of infecting pigs, and that a genetically similar strain isolated from pigs was capable of infecting non-human primates, suggesting a significant possibility that pigs could act as a zoonotic source of HEV (Meng et al. 1998). Since this study, many countries have noted the emergence of HEV cases. At this stage, it was thought that HEV was only endemic in developing countries (such as those in Asia, Africa and Central America), and that HEV cases in non-endemic areas were obtained through travel. However, studies such as those by Dalton et al. (2007) and Fraga et al. (2017) identified that indigenous cases of HEV were occurring in economically developed countries such as the UK and Switzerland. It is now widely accepted that pigs are a zoonotic source of HEV transmission to humans and may be at least partially responsible for increasing cases worldwide annually, though increased detection and awareness of HEV may also play a small role in the observed increase in cases. Outbreaks of HEV have directly been linked to pork product consumption, including an outbreak in Spain linked to consumption of wild boar (Rivero-Juarez et al. 2017), and another outbreak associated with consumption of a spit-roasted piglet in France (Guillois et al. 2015).

Consumption of pork products is now considered a significant risk factor for developing HEV infection, which is concerning considering the seroprevalence levels in European pigs (Said et al. 2014; Slot et al. 2017). Table 3 shows a non-exhaustive list of countries that have detected anti-HEV antibodies in pigs, and HEV RNA in pork products.

**Table 3:** A list of the seroprevalence levels of anti-HEV antibodies in pigs and the percentage of pork products found to be HEV positive by RT-PCR in those locations

Location	Pig sample population anti-HEV antibody seroprevalence	Percentage of tested foodstuffs HEV positive by RT-PCR
Brazil	63.6% of 357 pigs (Vitral et al. 2005)	1.7% of 118 slaughterhouse livers (Gardinali et al. 2012)
Canada	59.4% of 998 pigs (Yoo et al. 2001)	8.8% of 283 livers, 1.0% of 599 pork chops (Wilhelm et al. 2014)
		47.0% of 76 pork pâtés and 10.5% of 19 retail raw pork livers (Mykytczuk et al. 2017)
France	31.0% of 6565 pigs (Rose et al. 2011); 60% of 1034 pigs (Feurer et al. 2018)	4.0% of 3715 slaughterhouse livers (Rose et al. 2011)
		2.8% of 1034 slaughterhouse livers (Feurer et al. 2018)
		30.0% of 140 figatelli and fitone, 29.0% of 169 liver sausages, 25.0% of 55 quenelles or quenelle paste, 3.0% of 30 dried salted livers (Pavio et al. 2014)
		58.3% of 12 raw liver sausage (Colson et al. 2010)
Germany	49.8% of 1072 pigs (Baechlein et al. 2010)	4.0% of 200 retail livers (Wenzel et al. 2011)
		20.0% of 70 raw sausages and 22.0% of 50 liver sausages (Szabo et al. 2015)
Italy	45.1% of 2700 pigs (Mughini-Gras et al. 2017)	20.8% of 48 slaughterhouse livers (Di Bartolo et al. 2011)
		6.0% of 33 slaughterhouse livers (Di Bartolo et al. 2012)
		13.3% of 15 fresh liver sausages, 7.1% of 14 dried liver sausages (Di Bartolo et al. 2015)
Japan	57.9% of 2500 pigs (Takahashi et al. 2003)	1.9% of 363 retail livers (Yazaki et al. 2003)
Netherlands	89.0% of 417 organic pigs, 72% of 265 conventionally farmed pigs, 76% of 164 free range pigs (Rutjes et al. 2014)	6.5% of 62 commercial pork livers (Bouwknegt et al. 2007)
		12.7% of 79 livers, 70.7% of 99 liver sausages, 68.9% of 90 liver pâté samples (Boxman et al. 2019)
Spain	20.4% of 1441 pigs (de Oya et al. 2011)	6.0% of 93 sausages, 3.0% of 39 slaughterhouse livers (Di Bartolo et al. 2012)
Switzerland	62.3% of 1001 pigs in 2006, 53.8% of 999 pigs in 2011 (Burri et al. 2014)	1.3% of 160 slaughterhouse livers (Müller et al. 2017)
		11.8% of 102 raw liver sausages (Giannini et al. 2017)
		11.7% of 90 pork liver and raw meat sausages (Moor et al. 2018)
UK	92.8% of 629 pigs, with 20.5% viraemic at slaughter (Grierson et al. 2015)	10.0% of 63 sausages, 3.0% of 40 slaughterhouse livers (Berto et al. 2012)
USA	21.9% of 182 pigs (Owolodun et al. 2013)	11.0% of 127 retail liver (Feagins et al. 2007)

Considering Table 3, it is possible that the HEV prevalence in pork sausages may be over- or under-estimated by the fact that the sample sizes for some of these studies are relatively small. It is also important to note that different methods have been used between studies, some of which have been shown to be less sensitive for detecting HEV than others. Interestingly, the seroprevalence of HEV in pigs is significantly higher than the prevalence of HEV RNA in pork products in most cases; this discrepancy is expected as not all pigs would be viraemic at slaughter.

It has been found that HEV generally infects swine asymptomatically at an early point in their life (prior to 6 months of age (de Oya et al. 2011)), and that because of this, many pigs are seropositive at the time of slaughter (Grierson et al. 2015; Rose et al. 2011; de Oya et al. 2011). The slaughter age of pigs is normally slightly before 1 year of age. The transmission between pigs is suspected to be through a faecal-oral route and this is likely to be due to the high shedding that is seen in pig faeces and urine (Bouwknegt et al. 2009; Halbur et al. 2001). Infection with HEV early in life means that there is a lower chance of the pigs being viraemic at slaughter, and they are therefore less likely to be capable of HEV transmission to humans through the pork food chain. However, whether HEV causes life-long immunity in swine after recovery has been open to debate. In rhesus macaques and humans, the anti-HEV IgG antibodies (characteristic of long-term immunity) wane over a variable number of years until they are undetectable, and the period for which individuals may be IgG positive for varies (Arankalle et al. 1999; Lee et al. 1994). This may not be an issue in pigs as they are normally slaughtered before a year of age, so early life infections would likely allow immunity against subsequent HEV challenge over their lifetime. However, it has been shown that animals can be re-infected by different HEV strains (De Deus et al. 2008), and whether or not one strain can confer protective immunity to all other HEV strains has also been contested. It has been shown that after infection with one strain of G3 HEV, pigs developed some protective immunity against other strains within the same genotype, and also within G4 (Sanford et al. 2011). However, in rhesus monkeys, it was shown that infection with one strain from a genotype could not confer protective immunity to a strain in a different genotype upon subsequent challenge, and that in some cases, infection with a different strain from the same genotype could also not confer protective immunity (Huang et al. 2008). If there is genetic or environmental variation in host development of immunity to HEV, and some strains of HEV may not provide protection against others, then farms with multiple circulating HEV strains could be more likely to have viraemic pigs at the time of slaughter. This would therefore mean that there could be a higher likelihood of contracting HEV from undercooked pork products or food products containing raw pig components from these sources.

It has been reported that 21% of pigs in the UK tested positive for HEV RNA at the time of slaughter (Grierson et al. 2015), and that in the USA 6.3% of pigs from slaughterhouses were HEV RNA positive (Sooryanarain et al. 2020). As such, it is probable that the consumption of raw and undercooked pork products is acting as a transmission route of HEV to humans.

**Fig. 3:**
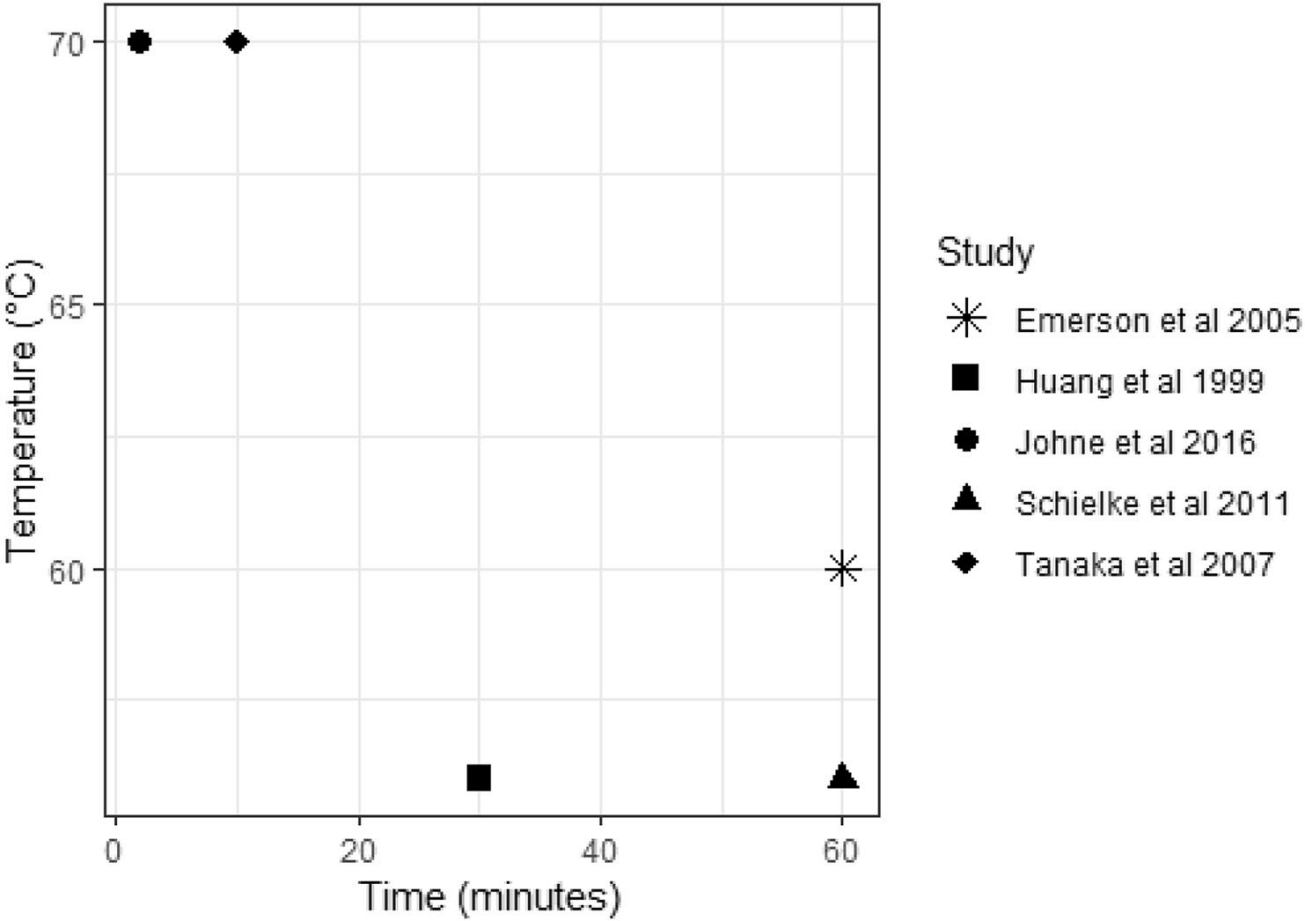
A summary of the reported thermal inactivation requirements for HEV from different studies. This graph summarises the observed HEV inactivation requirements for five different studies investigating the effect of heat treatment over time on HEV viability, with the highest reported inactivation requirements being 70 °C for 10 min, and the lowest being 56 °C for 30 min. Graph created in R studio

Table 4 summarises studies that have investigated the thermal inactivation of HEV in non-food matrix samples. Inactivation condition combinations found sufficient to inactivate virus in these studies are shown in Fig. 3. Results vary between the different studies, and several factors make comparison difficult. The studies use a variety of different units or expressions of reduction/inactivation. In the study by Huang et al. (1999), a temperature of 56 °C for 30 min was reported to completely inactivate the virus; however, the virus was only left to grow for a relatively short period of time (72 h). However, it was shown that HEV was still viable following similar heat treatments in cell culture studies with longer growth periods (Emerson et al. 2005; Tanaka et al. 2007). Schielke et al. (2011) used RNase treatment in an attempt to remove viral RNA that had broken from the capsid after heat treatment, assuming this would remove RNA from non-viable virus. However, it is unknown if any RNA from viable virus could have been lost during this treatment, and the standard deviations seen within the results were relatively large. Without a cell culture component, it is not possible to say with 100% certainty that remaining detected RNA was from viable virus. In addition to these limitations, it is known that HEV is difficult to culture effectively in vitro, often requiring large titres of virus to begin the culture, and therefore it is possible that the inactivation requirements for HEV have been under-estimated as treatments sufficient to eliminate infectivity in vitro may not completely eliminate in vivo infectivity. Some researchers have investigated different cell lines and strains of HEV which appear to be more efficiently cultured in vitro due to insertions within the HEV genome; however, culturing these strains still requires large titres of virus to begin the culturing process (10^6^ copies/ml) (Johne et al. 2014; Schemmerer et al. 2016).

**Table 4:** A summary of studies investigating thermal inactivation treatments for HEV in non-food matrix samples

Study	Cell culture or molecular detection?	Genotype	Heat treatment		Growth period	Inactivation/reduction
			Temperature (°C)	Time (min)		
Emerson et al. (2005)	Cell culture, HepG2/C3A cells	1 (strain Akluj^a^)	56	60	5–6 days	> 80% reduction
		1 (strain SAR55^b^)	56	60	5–6 days	~ 50% reduction
		2 (strain Mex14^c^)	60	60	5–6 days	96% reduction
Huang et al. (1999)	Cell culture, A549 cells	3 (strains G93-1*, G93-2*, G93-3*, G93-4*)	56	30	72 h	< 1.0 (TCID50/0.025 ml)
Johne et al. (2016)	Cell culture, A549/D3 cells	3 (strain 47832c^f^)	55	1	35 days	~ 1 log reduction in focus forming units
			70	2		“No infectious virus”
Schielke et al. (2011)	Molecular detection	3 (strain wbGER27^e^)	56	15	N/A	74.07% reduction
		3 (strain wbGER27^e^)	56	60	N/A	99.90% reduction
Tanaka et al. (2007)	Cell culture, PLC/PRF/5 cells	3 (strain JE03-1760F^d^)	70	10	35 days	“No infectious virus”
		3 (strain JE03-1760F^d^)	56	30	50 days	“Still infectious”

*Accession numbers unknown

^a^AF107909

^b^M80581.1

^c^KX578717.1

^d^AB437319.1

^e^FJ705359.1

^f^KC618403.1

It is possible that interactions of HEV with organic molecules in food matrices may cause the thermal inactivation temperature to be higher. Some studies have investigated the thermal inactivation of HEV within food stuffs, such as liver and pork sausage (summarised in Table 5). These studies have all taken different approaches to heat inactivation of the virus and the food types used, and therefore it is difficult to compare the results and form a definitive answer for the heat treatment required to inactivate the virus within foodstuffs. Feagins et al. (2007) identified that boiling or stir-frying infected pig liver to an internal temperature of 71 °C for 5 min could prevent infection when the liver was then fed to pigs. However, though the pigs were not infected by this oral dose, it is known that pigs commonly require a high dose of HEV to become infected through the faecal-oral route (Kasorndorkbua et al. 2004), of approximately 10^6^ genome copies (Andraud et al. 2013), and therefore it is possible that lower doses of active virus were still present in these food stuffs. The oral infectious dose for humans is unknown. Intravenous inoculation of pigs with the cooked foodstuffs as carried out by Barnaud et al. (2012) is likely to have provided a more accurate estimate of whether viable virus still existed, especially as intravenous inoculation of pigs has been reported to require much lower HEV doses to cause infection (Dähnert et al. 2018). Imagawa et al. (2018) reported similar inactivation requirements to Feagins et al. (2007). However, the limit of detection for the cell culture system was 10^4^–10^5^ genome copies, and therefore, as with the previous study, viable virus remaining in the minced pork may not have been detected in the cell culture system. In addition, the different food preparations between the studies may have influenced viral stability, as could the different strains of G3 HEV used. The initial viral titres used could also have influenced the results and explain why a longer treatment time was needed in some studies.

**Table 5:** A summary of studies investigating thermal inactivation treatments for HEV in foodstuffs

Study	Food stuff	Cooking method	Temperature (°C)	Time (min)	Measurement of inactivation	HEV inactivated?
Barnaud et al. (2012)	Pâté preparation (spiked with 10^8^ HEV genome copies)	Water bath	62	5	Intravenous administration to pigs	No
				20		No
				120		No
			68	5		No
				10		No
				20		No
			71	5		No
				10		No
				20		Yes
Feagins et al. (2008)	Pig liver (naturally infected)	Incubation	56	60	Oral administration to pigs	No
		Boiling	≥ 71 (internal)	5		Yes
		Stir fry	≥ 71 (internal)	5		Yes
Imagawa et al. (2018)	Minced meat (spiked with 10^10^ HEV genome copies)	Boiling or roasting	63	1	Cell culture	No
				5		No
				30		Yes
			65	1		No
				5		Yes
			70	1		No
				5		Yes

Further research is clearly required to investigate HEV inactivation within foods. This may require a more efficient cell culturing method and an assessment of different foods, cooking methods and HEV strains. However, taking the results of studies conducted in non-food and food (pork product) samples together, a conservative measure would be to cook pork products for longer than 20 min at temperatures higher than 72 °C.

In addition to the potential for HEV to survive some cooking processes, raw pork products are used in some consumables. Raw blood products are commonly used in ready-cooked foods such as processed ham (fibrinogen) and other blood proteins are used as food additives such as emulsifiers. Spray-dried plasma powder (SDPP) is also used in domestic and farm animal foods but this has some heat processing prior to use. SDPP is commonly fed to weaned piglets; however, a previous study reported no transmission of HEV in pigs fed spray-dried plasma products that were positive for HEV RNA (Pujols et al. 2014), and therefore the heating of spray-dried plasma may be sufficient to inactivate the virus present. However, as other porcine products are not subjected to heat processes before they are used, they could constitute a transmission risk to humans through use in food. A study conducted in 2017 found that of 36 liquid porcine products derived from blood, 33 were positive for HEV RNA, and seven of 24 spray-dried plasma products were also positive for HEV RNA (Boxman et al. 2017). This is especially significant when blood products from multiple animals are commonly pooled together, meaning that products from one viraemic animal could contaminate a batch and lead to widespread HEV transmission through many different food products.

Pork product consumption has been considered to be a major risk factor in the development of HEV due to the connection to foodborne outbreaks and the fact that HEV in pork products can reach high levels (e.g. 7 × 10^4^ genome copies/g in liver pâté in the Netherlands, Boxman et al. 2019). However, pigs are not the only animals consumed which can act as reservoirs for the virus.

## HEV in Other Land Animals and Animal Products

In addition to pigs, deer have been reported to be infected with HEV in many different countries (summarised in Table 6). It is important to identify the transmission risk that deer may have to humans, as HEV outbreaks have been directly linked to the consumption of raw deer meat. For example, in Japan, multiple people who had consumed raw Sika deer meat contracted HEV 6–7 weeks later (typical of the HEV incubation period), with the HEV sequence confirmed as 100% identical between the meat and infected patients (Tei et al. 2003). With the presence of HEV in deer being so variable between studies (perhaps due to study limitations such as sample size), further research is required to identify the level of active HEV infection within deer populations through larger prevalence studies. However, with the number of countries that have detected HEV in deer, and the occurrence of foodborne outbreaks from deer meat, deer could be acting as another reservoir for HEV.

**Table 6:** Summary of studies investigating the prevalence of HEV in deer

Study	Location	Deer species	ELISA observed seroprevalence	RT-PCR prevalence
Weger et al. (2017)	Canada	*Odocoileus virginianus* (White-tailed deer)	8.8%	ND
		*Odocoileus hemionus* (Mule deer)	4.5%	ND
		*Rangifer tarandus groenlandicus* (Barren-ground caribou)	1.7%	ND
		*Rangifer tarandus* (Woodland caribou)	5.2%	ND
Zhang et al. (2015)	China	*Cervus nippon* (Sika deer)	5.4%	ND
Anheyer-Behmenburg et al. (2017)	Germany	*Capreolus* (Roe deer)	0.0%	6.4%
		*Cervus elaphus* (Red deer)	0.0%	3.5%
Neumann et al. (2016)		*Cervus elaphus* (Red deer)	2.5%	3.7%
		*Capreolus* (Roe deer)	6.5%	0.0%
Reuter et al. (2009)	Hungary	*Capreolus* (Roe deer)	ND	12.2%
Di Bartolo et al. (2017)	Italy	*Cervus elaphus* (Red deer)	13.6%	11.0%
Sonoda et al. (2004)	Japan	*Cervus nippon* (Sika deer)	2.0%	0.0%
Matsuura et al. (2007)		*Cervus nippon* (Sika deer)	2.6%	0.0%
Tomiyama et al. (2009)		*Cervus nippon yesoensis* (Yezo deer)	34.8%	ND
Spancerniene et al. (2018)	Lithuania	*Capreolus* (Roe deer)	ND	22.6%
		*Cervus elaphus* (Red deer)	ND	6.7%
Medrano et al. (2012)	Mexico	*Odocoileus virginianus* (White-tailed deer)	62.7%	ND
Rutjes et al. (2010)	The Netherlands	*Cervus elaphus* (Red deer)	8.0%	15.0%
		*Capreolus* (Roe deer)	12.5%	0.0%
Boadella et al. (2010)	Spain	*Cervus elaphus* (Red deer)	10.4%	N/A^a^
Kukielka et al. (2016)		*Cervus elaphus* (Red deer)	12.9%	11.1%
Roth et al. (2016)	Sweden	*Capreolus* (Roe deer)	7.0%	0.0%
		*Cervus elaphus* (Red deer)	7.0%	0.0%

*ND* not done

^a^Did not test full sample population which were tested for seropositivity

There have been reports of HEV infections in cattle (*Bos taurus*) from China, where both antibody seroprevalence and HEV RNA of G4 have been identified in multiple studies. Hu and Ma (2010) showed the presence of G4 HEV RNA in 8.8% of cattle from Xinjiang Autonomous Region. A subsequent study then identified that 37.1% of tested dairy cows in Yunnan Province were HEV RNA positive, and that 100% of the HEV-positive cows were producing milk that contained HEV RNA (Huang et al. 2016). A further study in Shandong Province also found 3% of yellow cattle to be G4 HEV RNA positive, with 47% seroprevalent for anti-HEV antibodies (Yan et al. 2016). A study in Turkey identified HEV from G1, G3 and G4 in 20.3% of raw milk samples from various domestic animals (cows, sheep, goats, donkeys) (Demirci et al. 2019). Other studies investigating HEV in cattle have produced negative or mixed results; a study in Beijing, China identified 29.4% of cattle were seroprevalent for HEV, but no HEV RNA could be detected (Chang et al. 2009). A study in Burkina Faso also found 26.4% of 72 cattle to be seroprevalent for HEV (Ouoba et al. 2019). Another study in Germany testing 400 milk samples found no evidence of HEV RNA, although G4 HEV is much less commonly reported in Europe than in Asia (Baechlein and Becher 2017). Likewise, a study in Belgium also found no evidence of HEV RNA in cow milk or faeces (Vercouter et al. 2018). In the USA, Yugo et al. (2019) identified that of 983 cows, 20.4% were seroprevalent for anti-HEV antibodies; however, HEV RNA could not be detected in any of the cows. The authors concluded that this may have been because of an antigenically similar relative to HEV rather than due to HEV itself, which could be possible as G4 HEV is not thought to be endemic to the USA; however, this would call into question the specificity of ELISA assays and studies investigating HEV seroprevalence. Notwithstanding the significant number of studies with negative findings, these results are concerning as meat and dairy from cows are consumed worldwide by humans, and the possibility that cows could be a HEV reservoir could have a significant impact on our understanding of HEV transmission to humans. More research worldwide is therefore needed to identify which HEV genotypes are capable of infecting cattle, and to find the prevalence of HEV in cattle and dairy products. This will help to identify the risk of transmission of HEV from cattle to humans.

Goats have also been shown to be potential reservoirs for HEV infection, which is important due to goat meat, milk and cheese production. In Italy in 2016, 9.2% of goat faecal samples from six farms were found to be positive for HEV RNA, belonging to G3 strains which were highly related to strains found in pigs and humans (Di Martino et al. 2016). Also, in Yunnan province, China, Long et al. (2017) found 70.3% of goat faecal samples to be positive for HEV RNA, with milk samples from these animals also positive for HEV RNA. The strains obtained from these animals were from G4 HEV, and the Huang et al. (2016) and Long et al. (2017) studies highlight that farms with mixed animals may demonstrate a higher risk of HEV transmission. Another study in the Tai’an Region in China identified 4% of goat livers to be HEV positive, with G4 HEV that was similar to cow HEV detected in the same region (Li et al. 2017). In Turkey, 18.5% of goat milk samples were reported positive for HEV RNA (Demirci et al. 2019). Meanwhile in Burkina Faso, 28.4% of 81 goats were found to have anti-HEV antibodies (Ouoba et al. 2019). In the USA however, Sanford et al. (2013) suggested that a HEV-related agent was causing HEV in goats after discovering a seroprevalence of 16% but a lack of any HEV RNA; however, this again calls into question the accuracy of HEV ELISAs. In addition, no HEV RNA was detected by conventional RT-PCR in goats that had been experimentally infected with three different HEV strains from G1, G3 and G4 in this study; however, the sensitivity of this PCR may be lower than previously reported HEV PCR assays, as assays which target a larger amplicon are generally observed to have lower sensitivity (Debode et al. 2017), and qRT-PCR has generally been observed to be more sensitive for amplicon detection of HEV and other amplicon targets (Zhao et al. 2007; Zemtsova et al. 2015).

Dromedary camels have been implicated in the transmission of G7 HEV to humans. In one case, a patient who regularly consumed camel meat and milk contracted chronic G7 HEV after a liver transplant (Lee et al. 2016). This chronicity is likely to have been opportunistic and influenced by immunosuppressive medication to prevent organ rejection. In a separate paper, HEV was demonstrated to be seroprevalent in 23.1% of dromedary camels which originated in Sudan and Saudi Arabia (El-Kafrawy et al. 2020). Due to the recent discovery of this genotype of HEV, and its implication in human infection, further research is warranted to investigate how widespread camel HEV is within countries which regularly consume camel products to determine the risk such products may have for the foodborne transmission of HEV.

Rabbits and related species, e.g. hares, are also gaining increasing attention for their potential to transfer HEV to humans through consumption of meat. In France, five cases of rabbit HEV (defined within Orthohepevirus A, G3ra) were identified in confirmed HEV-positive patients out of 919 from 2015 to 2016 (Abravanel et al. 2017). Several countries have identified rabbits to be seroprevalent and RNA positive for HEV (Table 7). The number of observations and the apparent ability of humans to contract rabbit HEV suggest that it is a source of zoonotic HEV transmission. However, with most human cases worldwide belonging to other genotypes and sub-genotypes, rabbits are likely to be the cause of only a minority of cases. Studies have shown mixed results in terms of the ability of rabbits to carry other sub-genotypes of HEV. Zhang et al. (2017) have shown that though rabbits are capable of carrying sub-genotype 3ra, attempts to cause infections with another sub-genotype (3b) were unsuccessful; however, Hammerschmidt et al. (2017) identified a wild rabbit with HEV sub-genotype 3 g. Further research is therefore needed to identify which genotypes or sub-genotypes of HEV are capable of infecting rabbits. An interesting observation from the studies summarised in Table 7 is that concordance in HEV prevalence between different samples from the same animals is often lacking. For example, Burt et al. (2016) found that 60% of liver samples from 32 animals were HEV positive; however, only 16% of these 32 animals were faecally shedding the virus. It may therefore be wise to identify standardised testing methods worldwide for identification of infected animals, with a decision made on what samples to test and which assays are best to use, to avoid underestimating HEV prevalence.

**Table 7:** A summary of studies identifying HEV (genotype 3ra) in rabbits and hares

Country	Study	Seroprevalence	RNA prevalence
Burkina Faso	Ouoba et al. (2019)	60.0% of 100 rabbits, 52.6% of 19 hares	ND
Canada	Xie et al. (2017)	ND	5.0% of 63 companion rabbit faecal samples, 0.90% of 114 commercial rabbit faecal samples
China	Geng et al. (2011a)	54.6% of 119 farmed rabbits	7.0% of 119 farmed rabbit serum samples
	Geng et al. (2011b)	15.4% of 1094 farmed rabbits	2.0% of 1094 farmed rabbit serum samples
	Xia et al. (2015)	ND	5.0% of 492 rabbit faecal samples
	Li et al. (2020a)	ND	15.0% of 120 rabbit faecal samples
	Li et al. (2020b)	7.1% of 70 farmed rabbits	11.4% of 70 farmed rabbit faecal samples
France	Izopet et al. (2012)	ND	7.0% of 200 farmed rabbit bile samples, 23.0% of 205 wild rabbit liver samples
Germany	Eiden et al. (2016)	30.8% of 13 wild rabbits	30.8% of 13 wild rabbit serum samples
	Hammerschmidt et al. (2017)	37.3% of 164 wild rabbits, 2.2% of 669 wild hares	17.1% of wild rabbit serum samples, 0.0% of wild hare serum samples
	Ryll et al. (2018)	25% of 72 wild rabbits	34.7% of 72 wild rabbit liver samples
	Corman et al. (2019)	0.04% of 2389 wild hares	2.6% of 2389 wild hare serum samples
Italy	Di Bartolo et al. (2016)	3.4% of 206 farmed rabbits, 6.6% of 122 pet rabbits	0.0% of 7 IgG positive farmed rabbit serum samples, 0.0% of 122 pet rabbit serum samples
The Netherlands	Burt et al. (2016)	ND	23.0% of 35 petting farm rabbit faecal samples, 0% of 10 farmed rabbit liver and faecal samples, 60.0% of 32 wild rabbit liver samples and 16% of wild rabbit faecal samples
Poland	Bigoraj et al. (2020)	6.0% of 482 farmed rabbits	14.9% of 482 farmed rabbit liver samples
South Korea	Ahn et al. (2017)	ND	6.4% of 264 rabbit faecal samples
USA	Cossaboom et al. (2011)	36.5% of 85 rabbits	16.5% of 85 serum samples, 15.3% of 85 faecal samples

*ND* not done

Despite identifying that animal product consumption is a risk factor in the transmission of HEV, it is quite possible that there are other food transmission routes. One study in the Netherlands showed that though seroprevalence of HEV antibodies was higher in meat-eaters (22.8%), vegetarians still displayed a relatively high seroprevalence (13.8%) (Slot et al. 2017). This suggests that either they became HEV positive before becoming vegetarian through animal meat, or they were infected through other transmission routes. Figure 2 shows the known and theorised routes of HEV transmission. One transmission route, which is much more tightly controlled now, was the transmission of HEV through blood transfusion (Hewitt et al. 2014), which may be one way to explain the seroprevalence levels in vegetarians. Another explanation could be consumption of dairy products such as milk. It is also possible to contract HEV through organ transplant with an infected organ (Pas et al. 2012), and organ transplant-associated cases commonly result in chronic infections due to immunosuppression medication.

## Contamination of the Aquatic Environment

In addition to medical routes of transmission, it is important to consider the impact that HEV within animal farm run-off and sewage has on the aquatic environment. It has been suggested that human and farm sewage may have a part to play in other HEV transmission routes, potentially through farm run-off from animal slurry stores or application of animal slurry to crops and through contamination of surface waters used for irrigation and shellfish farms. Raw human sewage collected at 2-week intervals in 2014–2015 from a sewer which serves the whole of Edinburgh was found to contain HEV in 93% of the samples collected (Smith et al. 2016), and many other countries have also detected HEV in human sewage influent, such as Spain, Switzerland, Portugal and France (Clemente-Casares et al. 2003, 2009; Rodriguez-Manzano et al. 2010; Matos et al. 2018; Masclaux et al. 2013). This could therefore mean that when storm overflows discharge into water courses such as rivers and seas, HEV contamination can occur. Because there are many different types of wastewater treatment practices, and many combinations of practices between wastewater treatment plants, it is difficult to know which wastewater treatment plants will be more effective at removing viruses from wastewater. However, other viruses such as adenovirus and norovirus are commonly found in treated sewage (Bofill-Mas et al. 2006; Campos et al. 2016). It is therefore possible that in addition to storm overflows, inadequate treatment of sewage could result in HEV pollution of the aquatic environment, especially considering that HEV is a single-stranded RNA non-enveloped virus like norovirus. Pig farm and slaughterhouse sewage has also been found to be positive for HEV in multiple countries, for example, HEV RNA was detected in sewage from one of twelve slaughterhouses in Spain (Pina et al. 2000), whilst 75% of swine slurry samples collected from Italian pig farms were HEV positive (La Rosa et al. 2017), and both fresh swine faecal material and pooled stored slurry from pig farms in the USA were shown to contain HEV (Kasorndorkbua et al. 2005). Human sewage, pig farm run-off and abattoir outflows could also therefore be contaminating water courses with HEV, which is supported by studies in Italy, the Philippines and Cambodia, showing river water contamination with HEV (Iaconelli et al. 2015; Li et al. 2014; Baez et al. 2017; Rodriguez-Manzano et al. 2010).

Countries within the European Union must conform to EU regulations on how farm manure, animal carcasses and digestive tract content are processed, transported, stored, used as crop fertiliser, and disposed of. The sewage and wastewater that originates on farms must either be discharged to public sewers or treated in a sewage treatment plant on the farm before the effluent can be discharged to surface waters, and a permit is required for the processing and disposal of sewage and wastewater in this way. However, it is possible for farms within EU countries to use sewage and slurry that has been produced on a farm to be spread on crops at the same farm, without prior processing, for the sake of fertiliser or soil quality improvement (European Commission 2001). Accordingly, the United States also allows manure originating on one farm to be spread on crops from that farm (Environment Protection Agency 2020). However, manure use and farm practices are likely to be more diverse and potentially problematic in countries within Asia, Africa and South America.

Previous studies have shown that sewage treatment processes such as long-term fermentation and composting are likely to be capable of removing HEV from sewage (García et al. 2014). A study in Switzerland also identified HEV-positive influent samples from wastewater treatment plants, but no HEV-positive effluent samples, suggesting effective wastewater treatment using a cleaning and activated sludge process (Masclaux et al. 2013). However, Loisy-Hamon and Leturnier (2015) detected HEV in treated pig sewage samples from France that had been treated using one of sawdust composting, slurry dehydration or anaerobic digestion. Other studies have found that river water close to pig farms and pig processing plants had been contaminated with HEV, for example, in Scotland and Italy (Crossan et al. 2012; Idolo et al. 2013; Marcheggiani et al. 2015). Therefore, it is possible that leachate (liquid leaching from solids into the environment) from stored manure and yard run-off from farms and abattoirs may be polluting surface waters such as rivers. However, it is unknown whether all of the virus leaching into the environment is viable—for example, viral RNA detected in treated sewage may not indicate viable virus, but remaining RNA.

## Crop Contamination

Surface waters from sources such as rivers and groundwater are commonly used as crop irrigation sources throughout the world (Food and Agriculture Organisation 2011, 2016). Due to the potential contamination of such water with HEV and other pathogens from faecal matter (whether from human or animal sources), this could cause contamination of irrigated crops. Animal waste (that can potentially be contaminated with HEV as shown above) is also used as crop fertiliser for farms. A small number of studies have found some evidence of crop contamination with HEV. In France, two out of 230 herb and spice samples were positive for HEV RNA (Loisy-Hamon and Leturnier 2015), a study testing 125 lettuce samples from Greece, Serbia and Poland detected four positive samples (Kokkinos et al. 2012), and in Italy, six of 911 “pre-washed and ready to eat” vegetable samples tested positive for HEV RNA (Terio et al. 2017). Another study in four European countries (Czech Republic, Finland, Poland and Serbia) also detected HEV RNA in one frozen raspberry sample of 38 tested (Maunula et al. 2013). However, it is important to note that no foodborne outbreaks of HEV from contaminated crops have been reported, and the quantities of virus found on the crops is also low enough to call into question whether they would cause illness when consumed. It is also unknown whether the HEV RNA detected originated from viable virus.

## HEV in Bivalve Shellfish and Other Aquatic Animals

Bivalve molluscs are filter feeding organisms, meaning that they can accumulate and concentrate pathogens from their environment within their tissues. In the EU, bivalve shellfish are tested regularly for faecal contamination, using a faecal indicator, *Escherichia coli*, in accordance with food safety regulations. However, studies have shown that though it functions well as a bacterial faecal indicator, *E. coli* can be a poor indicator of the presence of faecally derived viruses. Lowther et al. (2012) found that norovirus RNA was present in 76.2% of total UK oyster samples from commercial harvesting areas, with 73.9% of those samples giving *E. coli* results compliant with the end product standard of ≤ 230 *E. coli*/100 g shellfish flesh. Norovirus within oysters is linked to human faecal pollution that has originated from storm overflows and CSOs, or sewage that has received insufficient treatment (Campos et al. 2013, 2016). CSOs release untreated sewage into surface water to prevent overflows within mains drainage, but outfall events can last for several hours or days and are often poorly monitored (Marine Conservation Society 2011). Considering that farm or abattoir run-off, combined sewer overflows, and inadequately treated sewage could be polluting watercourses with HEV, it is also possible for aquatic organisms, such as shellfish, to be affected by HEV contamination. Indeed, studies around the world have found HEV to be present within bivalve shellfish, and these are summarised in Table 8. The study by Rivadulla et al. (2019) also showed shellfish to have as much as 1.1 × 10^5^ RNA copies per gram of shellfish tissue, which is close to the pig ID50, but the human infectious dose is still unknown. It is important to note, however, that not all RNA found in the shellfish may have been associated with viable virus. To date, there have been no proven foodborne outbreaks of HEV from shellfish consumption, although an outbreak of HEV on a cruise ship was theorised to have been caused by consumption of shellfish on the basis of a retrospective risk analysis (Said et al. 2009).

**Table 8:** The presence of HEV in shellfish in different countries

Location	Study	Percentage of shellfish HEV positive
China	Gao et al. (2015)	17.5% of 126 shellfish samples^a^ of various species from production areas
Denmark	Krog et al. (2014)	0% of 29 mussel samples^a^ from 19 production areas
France	Grodzki et al. (2014)	0% of 286 shellfish samples^a^ of various species from two production areas
Italy	La Rosa et al. (2018)	2.6% of 384 shellfish samples^a^ of various species from production areas
Japan	Li et al. (2007)	6.3% of 32 Yamato-Shijimi clam samples^a^
Scotland	Crossan et al. (2012)	85.4% of 48 individual wild mussels
	O’Hara et al. (2018)	2.9% of 310 retail shellfish samples^a^ (mussels and oysters)
Spain	Mesquita et al. (2016)	14.8% of 81 mussel samples^a^ from a production area
	Rivadulla et al. (2019)	24.4% of 164 mussel, clam, and cockle samples^a^

^a^Where the study states that samples of shellfish were tested, it was either stated or assumed in each publication that each “sample” would have been formed by ten or more shellfish individuals and is therefore technically a pooled sample

HEV has also been found in other aquatic organisms, including dolphins, which present clinical symptoms of HEV infection. A study of 31 dolphins at the National Aquarium, Cuba, found that 32.2% of their dolphins were seroprevalent for HEV during two different studies (Villalba et al. 2017). The cause of the infections within the dolphins was unknown; however, it is possible that contamination of food items such as fish may be the cause, making an investigation of the presence of HEV in such animals important to determine whether there is any risk of HEV to humans from the consumption of fish. It may also be important to investigate the presence of HEV in aquatic mammals as they are used as a food source in some countries.

## Conclusion

In summary, the host range of HEV appears to be diverse, having been found within pig, deer, rabbit, cattle, goat and camels, amongst other animals. HEV has also been detected in shellfish meat as a result of contamination of their growing waters. Therefore, there is a risk of contracting HEV from undercooked products from these animals (although it is important to note that epidemiological evidence of foodborne transmission for many of these is currently lacking), and there is also potential for other livestock species to be unidentified hosts for the virus. Generally, foodstuffs containing raw meat or shellfish products are more likely to cause a foodborne infection than cooked foods or crops due to no thermal inactivation of the virus through cooking. Cooking in such a way that a minimum internal temperature of 72 °C is reached for at least 20 min is likely to completely inactivate any HEV present; however, this is likely to produce unwanted deterioration of organoleptic qualities in some risky food types, e.g. shellfish.

In addition to animal meat, milk from cows, sheep, goats, donkeys and camels has also been found to contain HEV in some countries, but studies investigating the presence of HEV in milk are much more limited. Because of this, the true risk of HEV transmission from animal milk is yet unknown and requires further research. However, if proven to be a prominent transmission route for the virus, a worrying consideration is that high-temperature short-time (HTST) pasteurisation of milk products, which is commonly used in the UK and USA, may be insufficient to reduce infectious HEV within milk, as generally the heat treatment used for HTST pasteurisation is only 72 °C for 15 s. Other pasteurisation methods, such as ultrahigh-temperature pasteurisation, which utilise treatments of around 135 °C for 2–4 s should be more capable of removing viable virus from milk products due to the higher temperature.

Though crops can also become contaminated with HEV, it seems that the risk of contracting HEV from them is much less likely, as confirmed outbreaks from crops have not been identified, and the HEV RNA prevalence and copies of viral RNA present are lower for these foods. However, it may be safe to conduct further research into the contamination of irrigation water, and the presence of HEV in crops from other countries to better assess the risk of contracting HEV from crop contamination.

It has also been shown that marine mammals can be infected with HEV, which is concerning both from an ecosystem and a seafood point of view. If marine mammals are becoming infected naturally, it could be possible that fish and other seafood also become contaminated or infected. Considering that shellfish in many countries have been found to be contaminated with HEV, this is perhaps something that warrants further investigation.

Due to mixed conclusions between and within countries about HEV presence within different hosts or matrices, it appears that there needs to be not only standardised and improved methods for the purpose of HEV detection, but also that further research through larger studies around the world is required to identify the full host range of HEV and the risk of each potential host to transmit the virus to humans (through food or other means). In particular, the suggestion that a HEV-related virus may be causing seroprevalence estimates to be higher than they genuinely are requires investigation.

Further studies identifying both the seroprevalence and the presence of HEV through ELISA and RT-PCR techniques, respectively (or similar techniques identifying RNA presence), would be best equipped to identify both the prevalence of the virus within animal populations and the amount of active infections within the populations at that point in time. However, sequencing technologies such as nanopore RNA sequencing within human and animal populations would also be useful to identify similarities between HEV sequences, enabling the identification of infection sources. Some studies investigating the evolution of the virus have already been performed, but are often biased by the large amount of HEV sequences derived from humans (Forni et al. 2018).

Investigations of HEV within food and environmental matrices using whole genome sequencing approaches have been limited so far due to the general observation of low genome copy numbers and fragmented HEV RNA within these matrices. However, techniques utilising methods such as multiplexing RNA extracted samples to obtain a full genome from multiple amplicons, followed by MinION next-generation sequencing, which has been successfully applied to sequencing of low levels of Zika virus (Quick et al. 2017), could be instrumental in future efforts to identify low levels of HEV in a variety of matrices, including foods.

Globally, HEV is an under-recognised viral threat, which causes an increasing case incidence annually. The best way to tackle a virus is to understand its sources and modes of transmission. Therefore, further research and better understanding of HEV will allow a better assessment of the risk that animal products and other foods may have in the transmission of HEV to humans. In turn, this may allow the introduction of legislative controls to prevent and control the spread of the virus.

## Supplementary Information

Below is the link to the electronic supplementary material.

Supplementary file1 (DOCX 1501 KB)

## Data Availability

All data are obtained from publicly available information.
