# Supplementary material for: The Foodborne Transmission of Hepatitis E Virus to Humans
Source: Food Environ Virol. 2021 Mar 18;13(2):127–45. doi: 10.1007/s12560-021-09461-5 (PMC8116281; doi:10.1007/s12560-021-09461-5)
Supplement: Supplementary file 1 — Supplementary file1 (DOCX 1501 KB) [file 12560_2021_9461_MOESM1_ESM.docx]

**Supplementary Data**

Supplementary data provided includes alternative versions of Fig 1 which are compatible for people with the conditions deuteranopia, protanopia and achromatopsia. The Fig 1 displayed in the article is compatible for the conditions protanomaly, deuteranomaly, tritanopia, tritanomaly, and achromatomaly, tested using colour blindness filters in GIMP.

*Supplementary data 1: Fig 1 compatible for the conditions deuteranopia and protanopia*

**Fig 1** The geographical distribution of HEV genotypes 1-4


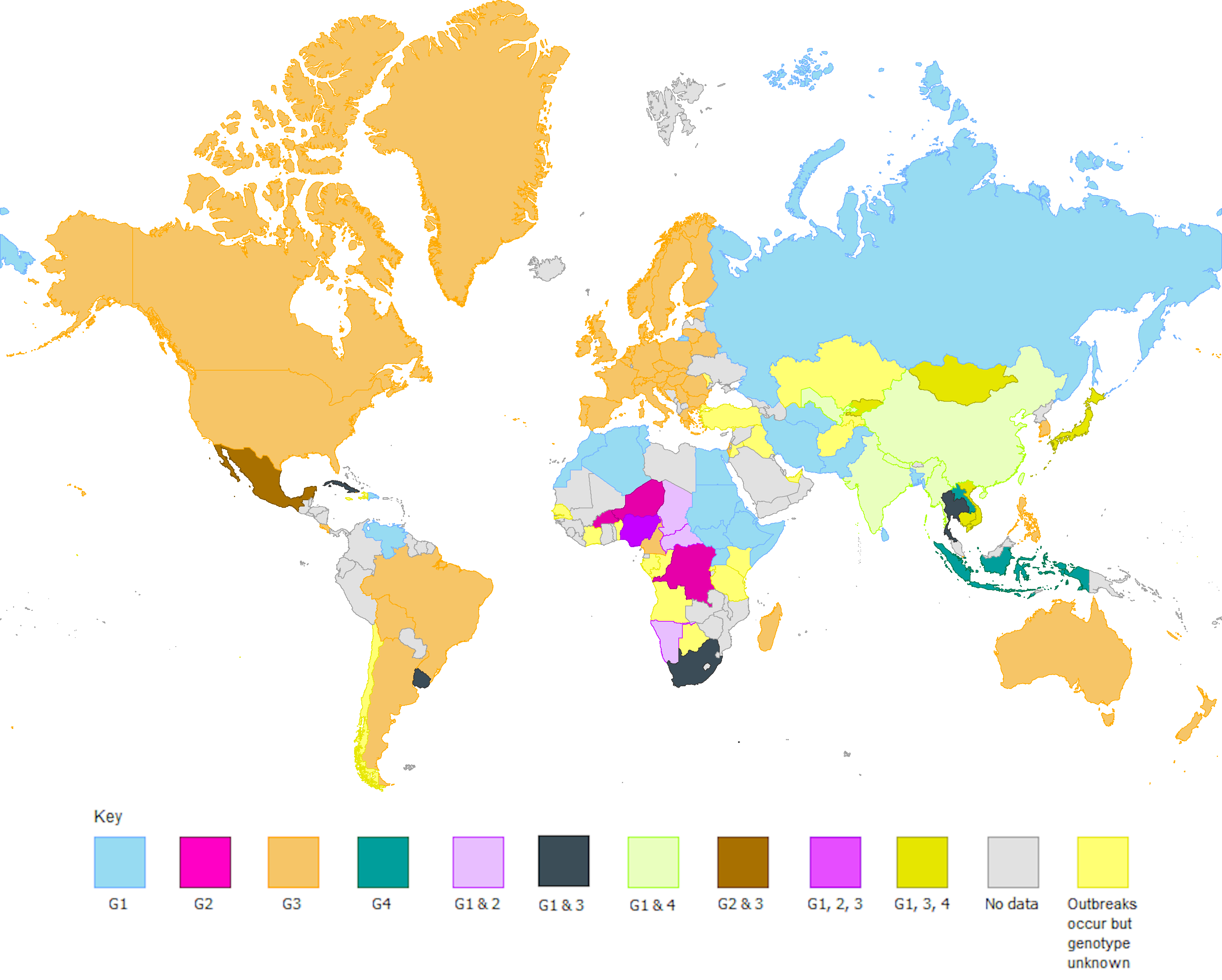


This figure shows the genotypes of HEV which are endemic to each country, where enough data was available. Maps created in ArcMap using the World Countries (generalized) layer package by esri_dm and visualised in GIMP.

*Supplementary data 2: Fig 1 compatible for the condition achromatopsia*

**Fig 1** The geographical distribution of HEV genotypes 1-4 (achromatopsia compatible)


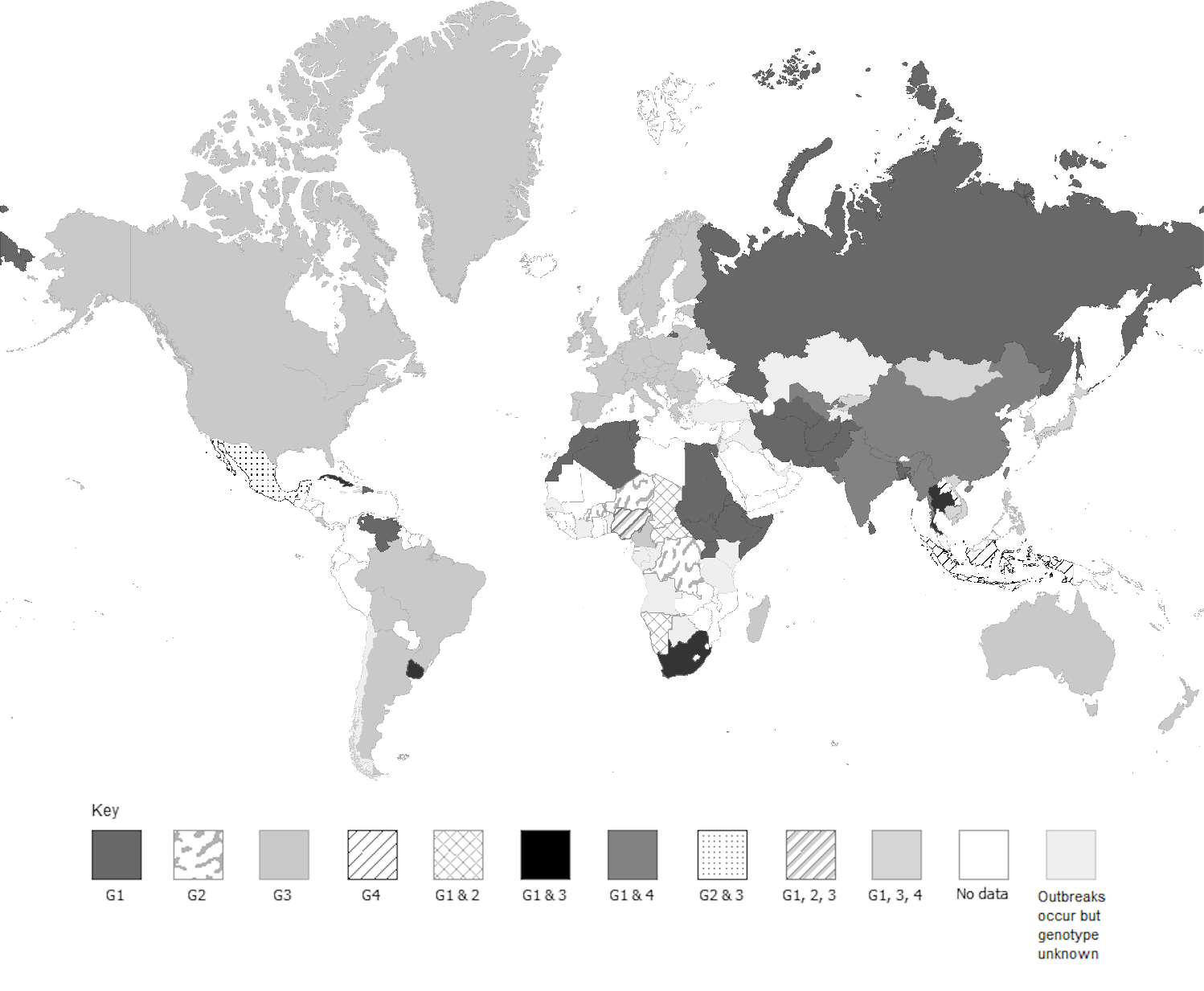


This figure shows the genotypes of HEV which are endemic to each country, where enough data was available. Maps created in ArcMap using the World Countries (generalized) layer package by esri_dm and visualised in GIMP.
